# Online interest regarding violent attacks, gun control, and gun purchase: A causal analysis

**DOI:** 10.1371/journal.pone.0207924

**Published:** 2018-11-28

**Authors:** Laura H. Gunn, Enrique ter Horst, Talar W. Markossian, German Molina

**Affiliations:** 1 Department of Public Health Sciences, Health Informatics and Analytics Program, University of North Carolina at Charlotte, Charlotte, NC, United States of America; 2 School of Public Health, Faculty of Medicine, Imperial College London, London, United Kingdom; 3 Universidad de los Andes (Uniandes), Facultad de administracion, Bogota, Colombia; 4 Department of Public Health Sciences, Stritch School of Medicine, Loyola University Chicago, Chicago, Illinois, United States of America; 5 Quantitative Research, Idalion Capital Group, London, United Kingdom; TNO, NETHERLANDS

## Abstract

**Background:**

Increased interest about gun ownership and gun control are oftentimes driven by informational shocks in a common factor, namely violent attacks, and the perceived need for higher levels of safety. A causal depiction of the societal interest around violent attacks, gun control and gun purchase, both synchronous and over time, should be a stepping stone for designing future strategies regarding the safety concerns of the U.S. population.

**Objective:**

Examine the causal relationships between unexpected increases in population interest about violent attacks, gun control, and gun purchase.

**Methods:**

Relationships among online searches for information about violent attacks, gun control, and gun purchase occurring between 2004 and 2017 in the U.S. are explained through a novel structural vector autoregressive time series model to account for simultaneous causal relationships.

**Results:**

More than 20% of the stationary variability in each of gun control and gun purchase interest can be explained by the remaining factors. Gun control interest appears to be caused, in part, by violent attacks informational shocks, yet violent attacks, although impactful, have a lesser effect than gun control debate on long-term gun ownership interests.

**Conclusions:**

The form in which gun control has been introduced in public debate may have further increased gun ownership interest. Reactive gun purchase interest may be an unintended side effect of gun control debate. U.S. policymakers may need to rethink current approaches to promotion of gun control, and whether societal policy debate without policy outcomes could be having unintended effects.

## Introduction

Gun control literature has attempted to quantify effects of gun regulation on crime rates, both within the United States [1–3] and around the world [4,5]. While this literature can inform gun control policies, it is also important to quantify information diffusion, interests, and emotions [6] to inform those policies [7]. Associated types of gun-related violence, which is more prevalent in the United States than in high-income countries [8], have also been thoroughly explored [9]. Scientific debate around benefits of gun control is as controversial as societal debate [10], with studies that support less stringent regulations [11] being immediately met with fierce peer-based rejections [12,13].

Internet searches provide a readily-accessible form of information about public opinion and interest, which could impact public health issues [14,15] and inform health policies [16,17]. This study aims to provide insights about relationships governing information diffusion and interests around topics of violent attacks, gun control, and gun ownership in the United States [1]. A shock is an unexpected alteration of a factor/variable of interest above its regular/steady levels, such as an increase in online searches about violent attacks that occurs concurrent with the actual violent attacks. The focus of this work is to assess the impact of shocks in interest about violent attacks on the information-seeking patterns about gun control and purchase. This will help identify impulse-response functions of the U.S. population to informational shocks regarding violent attacks, and also to assess the by-product impact of gun control debate on gun purchase interest.

Mass shootings’ impact on gun control and gun ownership debates oversize their relative importance, leading to more policy responses than other types of violence [18]. Interestingly, these policy responses have been more likely to reduce gun control in Republican-led legislatures and provided no impact in Democrat-led legislatures [18]. From a societal perspective, the literature on gun control has been prolific, across research perspectives, to report the potential benefits of enhanced gun control regulation [19–23], though with noted exceptions about its potential widespread benefits [24]. From an individual perspective, blame around mass shootings is oftentimes defined by the individual’s preconception around the gun control debate and actual firearm possession [25]. Media and social media, as key tunneling methods for the debate, have oftentimes led to polarization and entrenched ideological beliefs [26,27], rather than promoting a natural societal reaction to explore viable common grounds [28,29].

Anxiety driven by mass shootings oftentimes focuses the blame on guns across the ideological spectrum, offering an opportunity for policy change [29]. In contrast, gun ownership interest spikes after mass shootings [30]. This dual reaction points to the possibility that, while reactive societal interest around gun control may occur as a consequence of mass shootings, the synchronous impact of both events (mass shootings and gun control debate) leads to increased interests in gun ownership. While gun control debate has been theorized to be potentially and practically ineffective [31], statistically disentangling whether it is the mass shooting event or the oftentimes-concurrent gun control debate that drives this increased interest in gun ownership is a necessary step to understanding how societal advancements can occur. The main contribution of this manuscript is the extraction of the cross-factor causal impact among the factors of interest (informational interest about mass shootings, gun control, and gun ownership). This approach differs from previous research in that it is not descriptive in nature to demonstrate the potential (in)effectiveness of gun control debates or policies over time in the U.S., but inferential in showing that it is actually the current form of the gun control debate that may be causing increased gun ownership interest.

Google Trends is used to inform a time series model about societal information-seeking behaviors around gun-related violence from the perspectives of policy changes [32] and self-defense, in which gun control and gun purchase serve as proxies to behaviors and preferences for these two factors [33–35]. This approach, which is commonly used in econometrics, but relatively novel to public health analytics, explores potential causal relationships, in which shocks in internet searches about recent episodes of violence could induce shocks in gun control interest, and then shocks in the previous two factors could induce shocks in gun purchase interest.

## Methods

This study aims to describe the relationship between three unobservable variables within the U.S. population: (1) Interest about impactful violent attacks (VA); (2) Interest in gun control (GC); and (3) Interest toward gun purchase (GP). Since these factors are not directly observable without explicit disclosure (they are at most measurable through self-declaration, but impossible to extract retrospectively over long periods of time), internet searches about these topics were used as observable proxies for the historical interest levels at the population level.

### Data

It is assumed that Google searches provide a good representation of internet searches in the United States, since Google is the favored internet search engine [36,37], to the point that to search the internet has oftentimes been replaced by a newly-created verb, to google, added to the Oxford English Dictionary [38]. Google Trends, a tool provided freely by Google, offers a window into live data about global interests by individuals, and their evolution over time. Google Trends, as a way to access internet search data, has become a widespread tool for health research [15,39–44]. Google Trends was used to explore internet searches as proxies to the variables of interest [45]. Data collected from Google Trends consisted of multiple sets of 168 monthly observations over the period from January of 2004 to December of 2017, corresponding to Google-driven searches originating from U.S. IP addresses, for search terms provided in the next section.

### Study variables

In order to identify key search terms, an online search of news stories was conducted about the deadliest mass shootings in modern U.S. history [46] from the most popular [47] liberal and conservative media [48], extracting some of the common language used to define the first two groups below. The sets of search terms that follow are not intended to be all-inclusive, but to provide a basis to demonstrate the methodology, as well as provide initial insights over the nature of relationships between the different variables over time.

- VA: Google searches related to interest in violent attacks, comprised of any of the following key search terms: VA1 = {Shooting}, VA2 = {Shootings}, VA3 = {Gun Violence}, VA4 = {Massacre}, VA5 = {Terrorist Attack};
- GC: Google searches related to interest in gun control and regulation, comprised of any of the following key search terms: GC1 = {Gun Control}, GC2 = {Gun Laws}, GC3 = {Gun Rights}, GC4 = {Gun Safety}, GC5 = {Gun Regulations}; and
- GP: Google searches related to interest in gun purchase/ownership, comprised of any of the following key search terms: GP1 = {Gun Purchase}, GC2 = {Buy Gun}, GC3 = {Buy Weapon}, GC4 = {Concealed Weapon}, GC5 = {Concealed Carry}.

Word order in Google Trends is irrelevant, so both Gun Purchase and Purchase Gun are included within Gun Purchase. However, prepositions and articles are relevant, so search terms were defined as the minimum possible intersection of words in a common search (for example, Buy Gun includes searches such as How to Buy a Gun and Where to Buy a Gun). Plural versus singular forms of a word are also relevant, so both Shooting and Shootings were included, since they may reflect not only the nature of the violent attacks (single location versus potential multiple locations), but also intertemporal concerns (single shooting versus a perceived shooting spree). Note that violent attacks do not refer solely to those that occur with guns, but is a wider concept, while gun control and gun purchase explicitly seek to explore the online information-seeking reactions around those gun-related topics. We do not assume any particular motivation behind a search, nor political or ideological preferences around it.

**Fig 1:**
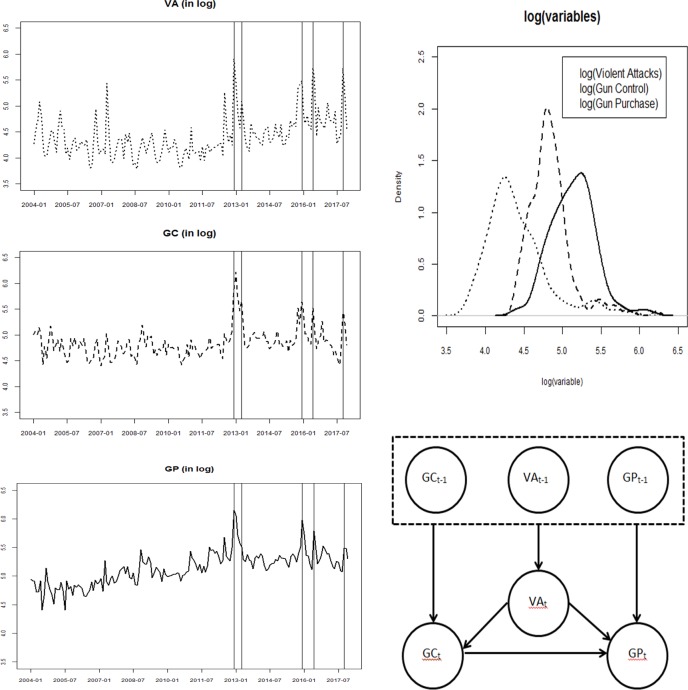
Time series representation (left), corresponding density representation (top right), and directed acyclic graphical representation of the relationships between study variables (bottom right), where t-1 represents the previous observation period for the variable and t represents the current observation period. Arrows represent the directionality of the causal relationships. Vertical lines mark the dates of several violent attacks (from left to right: Sandy Hook Elementary School, Boston Marathon, San Bernardino regional center, Orlando night club, and Las Vegas concert), which coincide with the peaks of these series.

All data is normalized in Google Trends with a common upper bound, and the combination variables (VA, GC, and GP) were normalized based on the key search term with the largest observed monthly searches within each set. This, in turn, has the advantage of scaling model parameters more homogeneously across experiments, as reflected in the top right panel of Fig 1, which shows the distribution of each combination variable (on the log scale). Google Trends additionally eliminates searches by the same individuals (i.e., originating from the same IP addresses) over short periods of time, though this unit of time is not explicitly defined by Google, nor whether it is constant or common for all searches.

The largest values in the data coincided with key events across all variables, outlining the endogenous nature of the relationships. The largest values of VA occurred in December of 2012 (Sandy Hook school shooting), June of 2016 (Orlando night club shooting) and October of 2017 (Las Vegas concert shooting). The largest values for GC occurred the months of and following the Sandy Hook shooting, as well as April of 2013 (Boston Marathon attacks). The largest values for GP also occurred the months of and following the Sandy Hook shooting and in December of 2015 (San Bernardino shooting). Time series plots for each of the combination variables, transformed to logarithmic scale, are presented in the left panel of Fig 1.

### Statistical model

The three variables of interest (i.e., online interests about violent attacks, gun control, and gun purchase) can have differing relationships amongst themselves. For example, certain violent attack information-seeking patterns may trigger gun control information-seeking patterns at the population level (i.e., a violent attack triggers gun control interest in part of the population). However, the reverse link would be less likely. This type of causal relationship can be naturally modelled through structural vector autoregressive (SVAR) time series models. The SVAR approach is commonly used in the econometric literature [49], and the resulting reduced form model allows for causal interpretability of the parameters of interest, rather than assessment of co-relationships.

The structural part refers to the natural constraints built within a standard vector autoregressive approach, which allow for causal analysis [50]. The SVAR approach allows for all three variables to be endogenous and related to each other both contemporaneously and in a lagged fashion. The model allows for autoregressive impacts, to capture temporal diffusion since the variables are correlated over time, in addition to contemporaneous relationships between the endogenous variables.

#### Model assumptions

All relationships were modelled at the population level only, which allows for individuals’ reactions to be notably different amongst themselves, and unknown to the researchers. Also, all relationships contained both a factor-specific level (α) and time-dependent trend component (μt), to reflect the baseline levels and long-term dynamics of interest in a topic, as well as demographic changes and higher internet penetration in society over time.

- Internet searches about violent attacks are not predictable: Violent attacks (and web-based searches about them) occur at random. They do not depend on debate regarding gun control (as long as that debate does not translate to substantial policy changes). They also do not depend on short term patterns in gun purchase (i.e., even if cumulative gun ownership was directly related to violent attack levels, it is assumed that monthly gun purchases are not).
- Gun control interest is influenced by violent attacks information-seeking: Shocks in violent attacks trigger reactions within the public. Some of those reactions relate to interest in more gun control, which is assumed to be linearly related to interest in violent attack information-seeking levels through a parameter β_21_. Gun control does not depend on contemporaneous gun purchase interest, but instead on cumulative gun ownership and existing gun ownership policies.
- Gun purchase interest is driven by information about violent attacks and gun control-related information: Interest in gun purchase, which is currently allowed legally in the U.S. through the second amendment, can occur for recreational purposes, crime-related reasons, and safety concerns [51]. It is assumed that the variability in gun purchase interest can also be explained by both violent attacks (as a fear-driven reaction) and increased interest/debate about gun control (preemptive purchases ahead of potential policy changes). These relationships are reflected linearly in the model through parameters β_31_ and β_32_, respectively.
- Variables follow an SVAR(1) model: No relevant seasonal components are assumed in the data. A Markovian structure is assumed, so that immediately previous values contain all necessary prior information about autoregressive trends. This assumption can be easily relaxed—for example by introducing seasonal components in gun purchases. Although a single autoregressive lag is an initial assumption in the model, Akaike Information Criterion (AIC = -10.32) tests [52] for best model fit validated this choice of a single lag. The series are all stationary according to the Augmented Dickie Fuller test (ADF: p≤0.01).

#### Model equations

The SVAR system of equations of interest has the following N = 3-dimensional autoregressive form:

$$A_{0}x_{t}=\alpha +\mu t+A_{1}x_{t-1}+e_{t}$$

where x_t_ is a 3x1 vector that includes the endogenous variables, now indexed at time t: VA_t_, GC_t_, and GP_t_. Define α and μ as 3x1 vectors of structural long-term level parameters, representing steady-level interest in the variables as well as long-term trend dynamics. {A_0_, A_1_} are 3x3 matrices of regression coefficients, and e_t_ is a vector of structural innovations (i.e., random noise), which are assumed to be temporally uncorrelated.

The matrix A_0_ reflects the Wold causal ordering [53] and has the following reduced form:

$$A_{0}=\left[\begin{array}{ccc} 1 & 0 & 0\\ {-\beta }_{21} & 1 & 0\\ -\beta_{31} & -\beta_{32} & 1 \end{array}\right]$$

which is implied by the following system of contemporaneous relationships, reflecting the model assumptions:

$${VA}_{t}=\alpha_{1}+\mu_{1}t+\varepsilon_{t}^{VA}$$

$${GC}_{t}=\alpha_{2}+\mu_{2}t+\beta_{21}{VA}_{t}+\varepsilon_{t}^{GC}$$

$${GP}_{t}=\alpha_{3}+\mu_{3}t+\beta_{31}{VA}_{t}+\beta_{32}{GC}_{t}+\varepsilon_{t}^{GP}$$

The bottom right quadrant in Fig 1 represents the SVAR relationships between the aforementioned variables in graphical form, where t-1 represents the previous observation period, and t represents the current observation period. The analysis was performed in R, using the vars package [54].

## Results

### Causal relationships and variance decomposition

Table 1 includes estimates for the b components of A_0_, which represent the contemporaneous causal linear relationships between the variables of interest (VA, GC, and GP). Positive relationships between VA and GC (b_21_ = 0.47) and between GC and GP (b_32_ = 0.36) were observed. The contemporaneous relationship between VA and GP was, surprisingly, significantly smaller (b_31_ = 0.07).

**Table 1:** Parameter estimates (and corresponding standard errors) and forecast error variance decomposition for the SVAR model (decomposition over 1, 2, 3, 6 and 12 months of shocks in the impulse variable [rows] over the response variables [columns]).

	Parameter estimates (standard errors)								Variance decomposition			
	Level	Trend	β (contemporaneous)			A_1_ (lagged)			Period	Forecast Error Variance Decomposition		
	α	μ*10^3^	VA	GC	GP	VA	GC	GP		VA	GC	GP
**VA**	2.48 (0.60)	3.11 (0.83)	1.00 (0.00)	0.00 (0.00)	0.00 (0.00)	0.34 (0.10)	0.39 (0.16)	-0.34 (0.17)	1	1.00	0.00	0.00
									2	0.86	0.05	0.08
									3	0.82	0.07	0.11
									6	0.80	0.09	0.11
									12	0.80	0.09	0.11
**GC**	1.37 (0.39)	0.14 (0.55)	0.47 (0.24)	1.00 (0.00)	0.00 (0.00)	-0.01 (0.06)	0.56 (0.11)	0.15 (0.11)	1	0.18	0.82	0.00
									2	0.18	0.81	0.01
									3	0.18	0.80	0.02
									6	0.17	0.79	0.04
									12	0.17	0.79	0.04
**GP**	2.02 (0.30)	2.37 (0.42)	0.07 (0.35)	0.36 (0.53)	1.00 (0.00)	-0.13 (0.05)	0.22 (0.08)	0.48 (0.08)	1	0.05	0.11	0.84
									2	0.04	0.18	0.78
									3	0.04	0.22	0.74
									6	0.04	0.22	0.74
									12	0.04	0.22	0.74

Table 1 also shows the variance decomposition for the SVAR relationship among the different factors for different lag times. After 12 months, for example, 20% of the long term, stationary variability in GC interest was explained through shocks in VA (17%) and GP (4%) interests. Similarly, after a year, 26% of the long term variability in GP interest was explained by shocks in VA (4%) and GC (22%) interests. These results indicate that, although there was a minor association between VA and GP, the majority of the impact on GP interest came from GC. While violent attack variations were important to explain variations of contemporaneous gun control interest, the variations of gun control interest were more relevant than violent attack variations when explaining changes in interest in gun purchase. Gun purchase interests, therefore, appear to be better explained by shocks in gun control interests.

**Fig 2:**
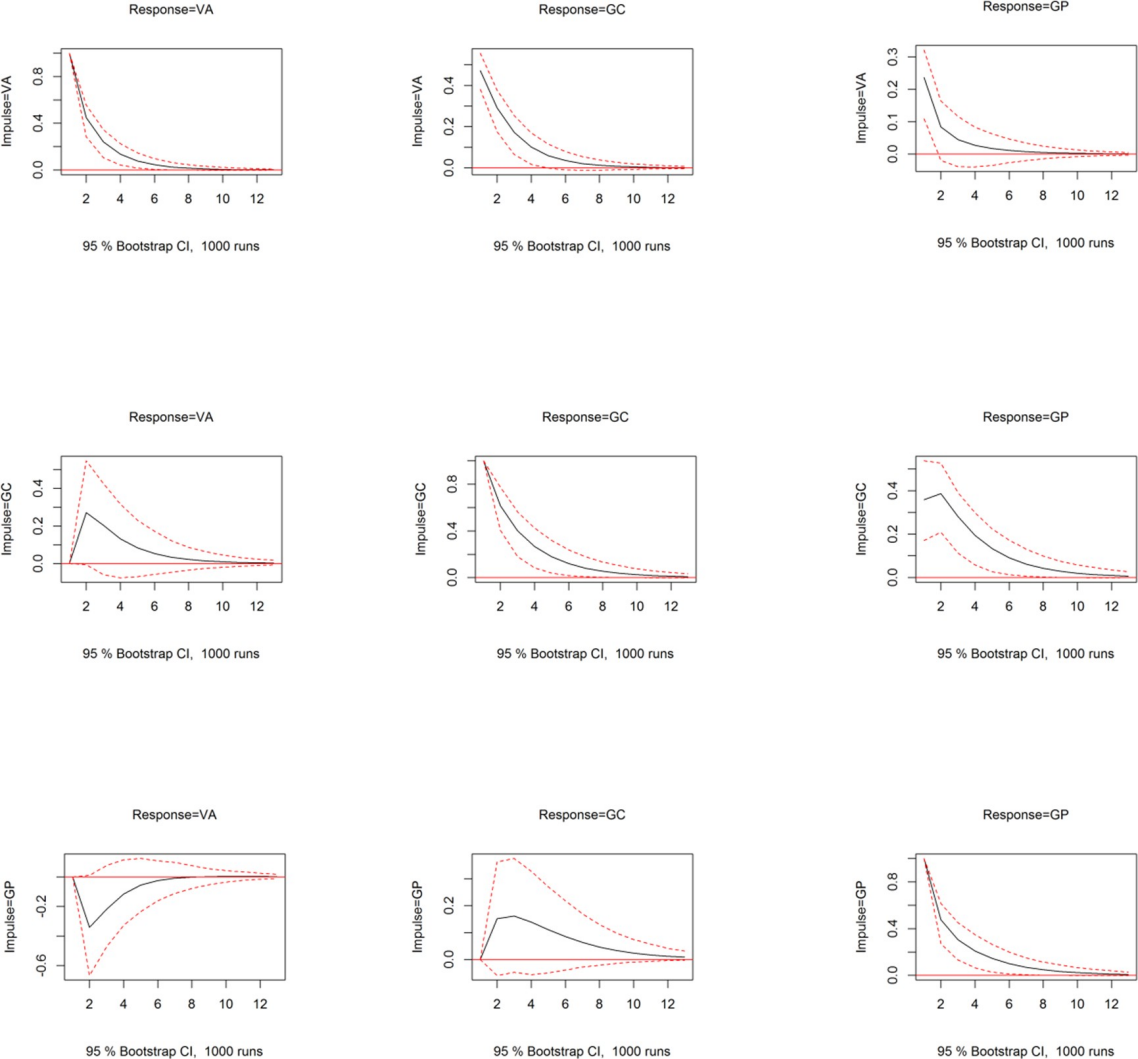
12-month impulse-response functions (solid line), with corresponding 95% confidence intervals (dotted lines), representing the impact of one standard deviation moves in the impulse variable on the corresponding response variable.

Finally, Table 1 presents estimates for the autoregressive components, which indicate not too dissimilar autoregressive behaviors among the variables, which is further reflected in the impulse-response functions in Fig 2.

### Impulse-response functions

The impact of shocks in each variable on all variables is best explored through their impulse-response functions [55]. Impulse-response functions represent the effect over time of independent shocks of one of the variables on all the variables in the system, keeping all other variables unchanged. Fig 2 shows a lattice graph with the impact of increases of one standard deviation in each variable (impulse) on each of the variables (response) over the following 12 months. Each figure contains the 95% bootstrap confidence interval for the impulse response function. Shocks in VA translated to statistically significant increased interest in GC, which lasted up to ten months before disappearing, but a statistically non-significant impact on GP over time. However, shocks in GC significantly impacted GP over the following ten to twelve months. Shocks in GP had small and non-significant effects on GC. The decay of shocks of a variable on itself was faster for VA and GC than that for GP, indicating that informational shocks about violent attacks died quickly, while those about gun purchase interest persisted for longer.

The results indicate that the main transmission of the shocks, other than from a variable to itself over time, occur from VA to GC and from GC to GP, with both relationships being significant at the 95% level, but not from VA to GP. Therefore, violent attacks informational shocks do not appear to be the main driver of gun purchase online interests, but instead shocks in gun control online interests are a more important driver. At the population level, when it comes to enhancing interests in gun purchases, it appears that the perceived risk of losing the right to have a weapon is a more powerful driver than the perceived risk of lack of safety associated with shocks driven by violent attacks.

### Search term decomposition

Table 2 contains results of running the model individually for each combination of key search terms comprising the composite variables used in the analysis (375 parameters corresponding to 5x5x5 = 125 submodels). In most cases, the relationships were strong, outlining that, although there are multiple ways in which the population can conduct searches about these topics, the underlying relationships behind these searches were widespread at the population level, and not overly-sensitive to the selection of key search terms.

**Table 2:** Estimates for the β coefficients, which represent the contemporaneous causal relationships between the study variables, for different search term combinations. The C variables represent the variables included in the model but not part of the corresponding pairwise relationship. Cell values represent the β coefficients for contemporaneous pairwise (causal) relationships between the variables in bold from the corresponding columns (causes) and rows (effects).

C		VA1	VA2	VA3	VA4	VA5	C		VA1	VA2	VA3	VA4	VA5	C		GC1	GC2	GC3	GC4	GC5
GP1	**GC1**	0.96	0.65	0.92	1.09	0.16	GC1	**GP1**	0.27	0.07	0.01	-0.03	-0.03	VA1	**GP1**	0.21	0.25	0.28	0.30	0.29
	**GC2**	0.64	0.34	0.39	0.40	0.05		**GP2**	0.08	0.00	0.05	0.07	-0.01		**GP2**	0.59	0.67	0.59	0.61	0.67
	**GC3**	0.46	0.31	0.42	0.57	0.02		**GP3**	0.35	0.13	0.16	0.11	0.01		**GP3**	0.27	0.30	0.26	0.33	0.37
	**GC4**	0.25	0.17	0.22	0.25	0.023		**GP4**	0.41	0.17	0.22	0.20	0.00		**GP4**	0.25	0.30	0.21	0.37	0.51
	**GC5**	0.40	0.19	0.31	0.25	0.06		**GP5**	0.39	0.18	0.21	0.24	-0.01		**GP5**	0.17	0.22	0.17	0.27	0.31
GP2	**GC1**	0.96	0.65	0.93	1.10	0.16	GC2	**GP1**	0.24	0.04	-0.04	-0.03	-0.03	VA2	**GP1**	0.09	0.14	0.19	0.18	0.17
	**GC2**	0.63	0.35	0.39	0.41	0.06		**GP2**	0.06	-0.07	-0.07	-0.03	-0.03		**GP2**	0.42	0.56	0.56	0.49	0.48
	**GC3**	0.44	0.33	0.44	0.60	0.03		**GP3**	0.27	0.08	0.08	0.07	-0.01		**GP3**	0.11	0.14	0.14	0.17	0.20
	**GC4**	0.25	0.18	0.23	0.26	0.03		**GP4**	0.30	0.11	0.12	0.12	-0.01		**GP4**	0.07	0.12	0.11	0.17	0.26
	**GC5**	0.40	0.21	0.32	0.26	0.07		**GP5**	0.26	0.09	0.08	0.11	-0.03		**GP5**	0.15	0.20	0.20	0.22	0.25
GP3	**GC1**	0.96	0.64	0.92	1.09	0.16	GC3	**GP1**	0.24	-0.13	-0.24	-0.21	-0.09	VA3	**GP1**	0.08	0.23	0.33	0.24	0.17
	**GC2**	0.63	0.34	0.39	0.40	0.05		**GP2**	0.05	-0.22	-0.12	-0.15	-0.08		**GP2**	0.38	0.71	0.59	0.54	0.42
	**GC3**	0.44	0.31	0.42	0.57	0.02		**GP3**	0.25	-0.06	-0.03	-0.09	-0.06		**GP3**	0.12	0.22	0.23	0.23	0.20
	**GC4**	0.27	0.16	0.21	0.24	0.01		**GP4**	0.34	0.00	0.04	0.02	-0.08		**GP4**	-0.18	0.03	0.04	0.03	0.06
	**GC5**	0.41	0.19	0.31	0.25	0.06		**GP5**	0.27	-0.03	0.03	0.00	-0.07		**GP5**	0.08	0.20	0.20	0.19	0.21
GP4	**GC1**	0.96	0.64	0.91	1.11	0.17	GC4	**GP1**	0.32	0.21	0.11	-0.03	-0.02	VA4	**GP1**	0.04	0.02	0.06	0.16	0.15
	**GC2**	0.64	0.34	0.39	0.40	0.05		**GP2**	0.17	0.12	0.02	-0.03	-0.02		**GP2**	0.29	0.27	0.41	0.47	0.45
	**GC3**	0.46	0.31	0.42	0.59	0.03		**GP3**	0.33	0.20	0.14	0.06	0.00		**GP3**	0.08	0.06	0.08	0.16	0.18
	**GC4**	0.26	0.16	0.21	0.25	0.02		**GP4**	0.32	0.19	0.13	0.07	0.00		**GP4**	0.19	0.17	0.22	0.33	0.38
	**GC5**	0.41	0.19	0.31	0.25	0.07		**GP5**	0.35	0.20	0.15	0.12	-0.01		**GP5**	0.02	0.04	0.05	0.13	0.15
GP5	**GC1**	0.96	0.66	0.92	1.09	0.16	GC5	**GP1**	0.30	0.13	-0.01	-0.01	0.00	VA5	**GP1**	0.03	0.04	0.13	0.13	0.13
	**GC2**	0.66	0.35	0.39	0.40	0.06		**GP2**	0.06	0.01	-0.08	-0.05	0.00		**GP2**	0.39	0.43	0.55	0.48	0.45
	**GC3**	0.47	0.33	0.43	0.58	0.03		**GP3**	0.29	0.13	0.07	0.04	0.01		**GP3**	0.08	0.09	0.12	0.15	0.17
	**GC4**	0.26	0.18	0.22	0.25	0.02		**GP4**	0.28	0.13	0.07	0.06	0.01		**GP4**	0.18	0.21	0.27	0.31	0.36
	**GC5**	0.41	0.21	0.31	0.25	0.07		**GP5**	0.30	0.14	0.09	0.10	0.00		**GP5**	0.05	0.09	0.10	0.14	0.16

The relationship between violent attacks and gun control (left block in Table 2) was strong regardless of the choice of gun purchase search terms (all 125 relationships explored were positive). For example, a model which only included VA2, GC1, and GP2 (i.e., Shootings, Gun Control, and Buy Gun as the search terms), indicated a relationship between VA2 and GC1 of b21 = 0.65. Similarly, the relationship between gun control and gun purchase (right block in Table 2) was consistently positive, with b32 = 0.42 in this example, and only 1 negative result out of 125 relationships explored). The relationship between violent attacks and gun purchase appeared weaker across the board, with most parameters around zero (b31 = 0 in this example).

## Discussion

This is the first study, to our knowledge, that uses internet searches as a proxy for interest in, and measurement of, intertemporal causal relationships between interest in violent attacks, gun control, and gun purchase at a population level. Results from the impulse-response functions show that a surge in interest about violent attacks induced subsequent surges in interest about gun control and gun purchase, but with a higher longer-term impact on gun control interest and much more subdued and non-significant impact on gun purchase interest. However, results show that heightened gun control interest appears to cause significant and lasting increases in gun purchase interest. Public interest in violent attacks can spark interest in self-protection in different forms, although evidence found in surveys about viewpoint changes related to specific events is limited [56]. Among possible reactions, two antagonistic options are the search for individual protection (gun purchase) and societal protection (gun control).

Additionally, gun control debate can spark fears in those who understand it not only as a threatened second amendment right, but also as a necessary means for self-protection. The time series SVAR model accounts for these contemporaneous causal relationships; and, population-based variations in interest were explored through Google searches around key search terms that reflect these societal actions and fears. Results show that there are strong relationships between shocks in information interest about violent attacks and reactive interest about gun control, but also that shocks in interest regarding gun control tend to have the opposite intended effect on gun purchase interest, enhancing it rather than taming it.

Since internet searches imply a perceived lack of sufficient knowledge about the topic, yet interest in it, it is important to note that the impact on gun purchase interest by gun control interest may be higher than observed. Individuals who do not need to conduct a search about gun purchase, either because they already own firearms or already know how to purchase them, would be less likely to be included amongst our data. Since individuals were not sampled at random in this analysis, it would be reasonable to consider those who own a gun, or possibly those who have knowledge of how to purchase one, as a subpopulation of those more likely to be against gun control (or at least some degree of it).

Finally, the impulse-response functions facilitate understanding the causal relationships and temporal length of the impact of shocks in each of the variables. While interest regarding violent attacks seems to decay quicker as information is absorbed, gun control and gun purchase interest appear to have high persistence to its own shocks. At the population level, once information is sought about gun control or gun purchase, the interest appears to be highly persistent.

### Strengths and limitations

Open discussion about gun control and gun ownership faces significant sampling and funding issues. For example, funding from CDC or NIH is highly restricted around this topic, and, as a consequence, its research has become a self-funded effort rather than one aligned with its relative relevance comparatively to other health issues with similar mortality rates [57–59]. Additionally, there are no databases on firearm ownerships in the U.S. (impossible under federal law [60]), making direct research accounting for ownership unfeasible without self-declaration.

Direct cross-sectional surveying about self-declared gun purchase intentions has shown little impact of some violent attacks on gun purchase, but with no assessment of biases [56]. A more global and dynamic survey, performed on a monthly basis and on the scale of the data used from Google Trends, would be logistically impossible as a retrospective analysis.

A prospective study would be theoretically feasible, though it would need a staged (stepped-wedge) approach to ensure that the sample continuously mimics the population of interest in the same way Google Trends searches do (a multi-year survey from a fixed sample would be biasing the results by age). However, it would suffer with strong limitations, as it would be financially prohibitive [57,58], potentially biased during key times when shocks in information occur, and it would take an unreasonable length of time to capture the same amount of information. Instead, this study utilized the perceived privacy of internet searches, which may not be available through direct contact/survey of individuals.

This is an observational study. Individuals contained within the population of interest are not randomized and share a common trait (i.e., subpopulation of Google-based information-seekers). However, the level of penetration of the internet in the population [61], and of Google as a search engine [36], can help alleviate potential sampling biases and is widely used to explore population health issues [15,39–44].

Some individuals may already possess knowledge about gun purchasing options, not needing to conduct a web-based search. This study considered three variables, one of which is most likely relevant to the period under consideration (i.e., enhanced interest about violent attacks), while the remaining two variables (i.e., gun control and gun purchase), would more likely allow for cumulative knowledge, particularly gun purchase. This may result in underestimation of gun purchase interests, since gun ownership interests would be comprised of new interests and prior interests with information already acquired.

This study does not explore the effect of cumulative gun purchase interest or gun control interest on violent attacks. The variables in this work relate to active searches of information. If an individual accesses a story on a news webpage that shows information about any of the constructs without having gone through a Google search, this is not defined in the study as an active search and will not appear in the data.

Finally, this approach represents societal reactions, rather than individual behaviors. Therefore, results are applicable at the population level, and are not appropriate to describe the relationship between these factors at the individual level.

### Public health implications

Although gun control debate may be a necessary means for social buy-in, and to achieve higher penetration of interest about increased regulation of firearms, its implementation may also need to be rethought. Shocks of information related to heightened debate around the gun control topic, oftentimes driven by stakeholders in the topic, such as politicians or media, may be triggering fears within a portion of the population who may be undecided or borderline about gun ownership (i.e., those who do not already own a gun and may be more likely to search the internet about gun purchase). Gun control supporters may find themselves in a situation in which policy changes will not happen without societal debate, but societal debate may be increasing gun ownership interest. Gun control debate resulting in no policy changes may be increasing interest, as results suggest, and potentially also desire, for gun ownership. In fact, results indicate that long-term gun purchase interest are affected more significantly by shocks in gun control societal interest than shocks in interest in violent attacks, which have a contemporaneous effect, but a lesser long-term impact than gun control informational shocks.

This paper offers a window into understanding links between key events around violent attacks and the population’s reactions to them, as well as the by-product debate around gun control and its effects on the population. While it is impossible to map interests to behaviors at the scale of this analysis, this study provides first insights into causal effects around key drivers to U.S. gun purchase interests.

Common ground solutions are needed in Public Health topics as polarized as gun control and ownership regulation [28]. However, finding that common ground requires not only exposition of perspectives around optimal solutions from each side of the ideological spectrum, but offering perceived solutions to anxieties and fears held by those who disagree.

Media and social media have become a reflection of a polarized society [27], and dialogue around common grounds has been replaced with clustering of policies around entrenched ideological beliefs, with media promoting a larger separation of political views, rather than promoting the search for a common ground [26]. The current form in which gun control debate is occurring appears to have been fruitless both in terms of policy changes [18] and in terms of societal changes [30]. In a country where demand for gun control could be very high due to its gun violence statistics [23] and its societal reaction after each mass murder [29], it is of most relevance to question the current form in which that debate is being promoted, and whether it has been effective or counterproductive. Gun control promoters believe that challenging the gun policy status quo is needed for safety and Public Health advancement [23]. Challenging the form in which gun control debate is being introduced may also be needed.

This manuscript constitutes a contribution to the assessment of the impact of current gun control debate. Gun control informational interest is found in this paper to be a key factor driving informational interest around gun ownership, surpassing the interest generated by the often-concurrent mass shootings. Gun control academic research about its societal benefits has been as overwhelming as its ineffectiveness to change the status quo. Anxiety about losing the right-to-own appears to be a strong driver of increased gun ownership interests, and those individuals may perceive the current form of gun control debate as a zero-sum game, where any change will bring a worsened outcome. Future gun control research should focus on providing solutions to individuals’ anxieties and fears, so that societal change can take place. Policy proposals and an effective gun control debate could be founded on providing viable solutions to the underlying causes driving reactive gun purchase interests, rather than further marketing entrenched perspectives [26]. Framing a safe, truly inclusive debate away from arguments that may induce additional anxieties and fears, in order to find common grounds and adaptive solutions, will require both sides of the spectrum to move away from their maximalist viewpoints.
